# dbTMM: an integrated database of large-scale cohort, genome and clinical data for the Tohoku Medical Megabank Project

**DOI:** 10.1038/s41439-021-00175-5

**Published:** 2021-12-10

**Authors:** Soichi Ogishima, Satoshi Nagaie, Satoshi Mizuno, Ryosuke Ishiwata, Keita Iida, Kazuro Shimokawa, Takako Takai-Igarashi, Naoki Nakamura, Sachiko Nagase, Tomohiro Nakamura, Naho Tsuchiya, Naoki Nakaya, Keiko Murakami, Fumihiko Ueno, Tomomi Onuma, Mami Ishikuro, Taku Obara, Shunji Mugikura, Hiroaki Tomita, Akira Uruno, Tomoko Kobayashi, Akito Tsuboi, Shu Tadaka, Fumiki Katsuoka, Akira Narita, Mika Sakurai, Satoshi Makino, Gen Tamiya, Yuichi Aoki, Ritsuko Shimizu, Ikuko N. Motoike, Seizo Koshiba, Naoko Minegishi, Kazuki Kumada, Takahiro Nobukuni, Kichiya Suzuki, Inaho Danjoh, Fuji Nagami, Kozo Tanno, Hideki Ohmomo, Koichi Asahi, Atsushi Shimizu, Atsushi Hozawa, Shinichi Kuriyama, the Tohoku Medical Megabank Project Study Group, Masayuki Yamamoto, Michiaki Abe, Yayoi Aizawa, Yuichi Aoki, Koichi Chida, Inaho Danjoh, Shinichi Egawa, Ai Eto, Takamitsu Funayama, Nobuo Fuse, Yohei Hamanaka, Yuki Harada, Hiroaki Hashizume, Shinichi Higuchi, Sachiko Hirano, Takumi Hirata, Masahiro Hiratsuka, Atsushi Hozawa, Kazuhiko Igarashi, Jin Inoue, Noriko Ishida, Naoto Ishii, Tadashi Ishii, Mami Ishikuro, Kiyoshi Ito, Sadayoshi Ito, Maiko Kageyama, Fumiki Katsuoka, Hiroshi Kawame, Junko Kawashima, Masahiro Kikuya, Kengo Kinoshita, Kazuyuki Kitatani, Tomomi Kiyama, Hideyasu Kiyomoto, Tomoko Kobayashi, Eiichi Kodama, Mana Kogure, Kaname Kojima, Sachie Koreeda, Seizo Koshiba, Shihoko Koyama, Hisaaki Kudo, Kazuki Kumada, Shigeo Kure, Miho Kuriki, Shinichi Kuriyama, Yoko Kuroki, Norihide Maikusa, Satoshi Makino, Hiroko Matsubara, Hiroyuki Matsui, Hirohito Metoki, Takahiro Mimori, Naoko Minegishi, Kazuharu Misawa, Masako Miyashita, Satoshi Mizuno, Hozumi Motohashi, Ikuko N. Motoike, Satoshi Nagaie, Masato Nagai, Fuji Nagami, Masao Nagasaki, Sachiko Nagase, Naoki Nakamura, Tomohiro Nakamura, Naoki Nakaya, Keiko Nakayama, Akira Narita, Ichiko Nishijima, Takahiro Nobukuni, Kotaro Nochioka, Taku Obara, Soichi Ogishima, Noriaki Ohuchi, Gervais Olivier, Noriko Osumi, Hiroshi Otsu, Akihito Otsuki, Daisuke Saigusa, Sakae Saito, Tomo Saito, Masaki Sakaida, Mika Sakurai-Yageta, Yuki Sato, Yukuto Sato, Atsushi Sekiguchi, Chen-Yang Shen, Tomoko F. Shibata, Ritsuko Shimizu, Kazuro Shimokawa, Matsuyuki Shirota, Junichi Sugawara, Kichiya Suzuki, Yoichi Suzuki, Shu Tadaka, Makiko Taira, Takako Takai-Igarashi, Yuji Takano, Yasuyuki Taki, Gen Tamiya, Osamu Tanabe, Hiroshi Tanaka, Yukari Tanaka, Shunsuke Teraguchi, Takahiro Terakawa, Teiji Tominaga, Hiroaki Tomita, Akito Tsuboi, Naho Tsuchiya, Ichiro Tsuji, Masao Ueki, Akira Uruno, Nobuo Yaegashi, Junya Yamagishi, Yumi Yamaguchi-Kabata, Chizuru Yamanaka, Riu Yamashita, Jun Yasuda, Junji Yokozawa, Kazunori Waki, Makoto Sasaki, Junko Akai, Ryujin Endo, Akimune Fukushima, Ryohei Furukawa, Tsuyoshi Hachiya, Kouhei Hashizume, Jiro Hitomi, Yasushi Ishigaki, Shohei Komaki, Yuka Kotozaki, Takahiro Mikami, Motoyuki Nakamura, Naoyuki Nishiya, Satoshi Nishizuka, Yoko Nomura, Kuniaki Ogasawara, Hideki Ohmomo, Shinichi Omama, Ryo Otomo, Kotaro Otsuka, Kotaro Oyama, Kiyomi Sakata, Ryohei Sasaki, Mamoru Satoh, Namie Sato, Atsushi Shimizu, Yu Shiwa, Yoichi Sutoh, Nobuyuki Takanashi, Noriko Takebe, Fumitaka Tanaka, Ryoichi Tanaka, Kozo Tanno, Tomoharu Tokutomi, Kayono Yamamoto, Fumio Yamashita, Nobuo Fuse, Teiji Tominaga, Shigeo Kure, Nobuo Yaegashi, Kengo Kinoshita, Makoto Sasaki, Hiroshi Tanaka, Masayuki Yamamoto

**Affiliations:** 1 Tohoku Medical Megabank Organization, Tohoku University, Sendai, Japan; 2 Graduate School of Medicine, Tohoku University, Sendai, Japan; 3 Advanced Research Center for Innovations in Next-Generation Medicine, Tohoku University, Sendai, Japan; 4 Tohoku University Hospital, Tohoku University, Sendai, Japan; 5 International Research Institute of Disaster Science, Tohoku University, Sendai, Japan; 6 Iwate Tohoku Medical Megabank Organization, Iwate Medical University, Morioka, Japan; 7 Graduate School of Information Sciences, Tohoku University, Sendai, Japan

**Keywords:** Databases, Medical research

## Abstract

To reveal gene-environment interactions underlying common diseases and estimate the risk for common diseases, the Tohoku Medical Megabank (TMM) project has conducted prospective cohort studies and genomic and multiomics analyses. To establish an integrated biobank, we developed an integrated database called “dbTMM” that incorporates both the individual cohort/clinical data and the genome/multiomics data of 157,191 participants in the Tohoku Medical Megabank project. To our knowledge, dbTMM is the first database to store individual whole-genome data on a variant-by-variant basis as well as cohort/clinical data for over one hundred thousand participants in a prospective cohort study. dbTMM enables us to stratify our cohort by both genome-wide genetic factors and environmental factors, and it provides a research and development platform that enables prospective analysis of large-scale data from genome cohorts.

## Databases: Integrating megadata for disease research

A database integrating 1.3 trillion genome cohort data entries from 157,191 individuals in Japan will facilitate research into the gene-environment interactions underlying common diseases. The Tohoku Medical Megabank integrated database called dbTMM was developed by Soichi Ogishima, Masayuki Yamamoto and colleagues at Tohoku University in Japan. It incorporates the genome, metabolome, proteome, clinical, sociodemographic, lifestyle and environmental data from 84,073 adults, and 73,529 pregnant women and their families, including children. Blood and urine samples were collected from participants and analysed, then obtained genome/multiomics data were stored in dbTMM. Users can stratify the entire population into smaller populations based on multiple data variables, including whole genome variants, to search for statistically significant differences that might warrant further research. The dbTMM is expected to help clarify the genes and gene-environment interactions underlying common diseases and improve disease risk prediction.

## Introduction

Genome-wide association studies (GWASs), a genome-wide approach for identifying genetic variants associated with a trait^1^, have accelerated investigations of the genetic architecture of complex diseases. However, in common diseases, most genetic variants identified account for only a small fraction of the total phenotypic variation and cannot account for most of the heritability of diseases and phenotypes^2^. The proportions of heritability explained for common diseases are low, and rare variants or environmental factors modifying genetic risk through gene-environment (G × E) interactions are expected to explain this missing heritability.

To reveal the genes and gene-environment interactions underlying common diseases and estimate the risk of common diseases, prospective cohort studies have been conducted to characterize genes, exposures and risk factors before disease onset^3^. Several large-scale prospective cohort studies of gene-environment are underway, including the UK Biobank and All of Us. In Japan, in the Tohoku Medical Megabank (TMM) project^4^, we conducted a population‐based adult cohort study named “the TMM Community‐Based Cohort study (TMM CommCohort study)”^5^ and a birth and three‐generation cohort study named “the TMM Birth and Three‐generation Cohort study (TMM BirThree Cohort study)”^6^.

The TMM CommCohort study has been conducted in Miyagi and Iwate Prefectures, and the TMM BirThree Cohort study has been conducted in Miyagi Prefecture. More than 150,000 participants were successfully recruited and investigated in the baseline studies from 2013 to 2017, and their collected specimens have been stored in a biobank^7^. To clarify genes and gene-environment interactions in common diseases and develop risk scores for them, our biobank has conducted genome and multiomics analyses. Our biobank is an “integrated” biobank, having both biobank and analytical facilities for genomic^8^ and multiomics analysis^9^ to provide genome and multi-omics data of common interest instead of distributing the raw specimens. That is, our integrated biobank provides not only cohort/clinical data but also genome/multiomics data in addition to biospecimens from participants. We have developed an integrated database called “dbTMM” that incorporates both the individual cohort/clinical data and the genome/multiomics data of 157,191 participants.

The UK Biobank, All of Us, other biobanks and databases have thus far published catalogs and statistics for each data item, such as the UK Biobank Data Showcase (https://biobank.ndph.ox.ac.uk/showcase/) and the All of Us Data Browser (https://databrowser.researchallofus.org/); regarding genome data in particular, gnomAD (https://gnomad.broadinstitute.org/)^10^, TogoVar (https://togovar.biosciencedbc.jp/) for Japanese genome data, jMorp (https://jmorp.megabank.tohoku.ac.jp/)^11^ for multiomics data in the TMM project, and MGeND (https://mgend.med.kyoto-u.ac.jp/)^12^ for Japanese genomic and clinical information have been published. Researchers can look up statistics for each data item stratified by age and by sex but cannot browse data narrowed down by combining the conditions of multiple variables. In particular, the genome data are too large to search by variant. To search genome data on a variant-by-variant basis and to precisely stratify the cohort population by using gene variant and environmental data, we need to establish an integrated database. The ability to reveal gene-environment interactions and estimate the risk for common diseases depends largely on the ability to stratify a population into subpopulations with sufficiently distinct risks^13^. Drug development is also becoming dependent on genetic evidence, which increases the probability of success in Phase II and III clinical trials as well as approval by more than twofold^14,15^. Therefore, we have established dbTMM. dbTMM is the first database to store individual whole-genome data on a variant-by-variant basis and cohort/clinical data for over a hundred thousand participants in a prospective cohort study, which allows us to stratify our cohort by both genome-wide genetic factors and environmental factors. dbTMM allows researchers to generate hypotheses and begin assessing the potential of TMM data for their studies.

The underlying rationale for prospective genome cohorts is that to elucidate the mechanism of disease pathogenesis, it is necessary to recruit a population of healthy individuals, follow them for more than 20 years, collect all of their environmental exposure and genetic factor data before the onset of disease in a nonhypothesis-driven manner, and then prospectively analyze all of participants by deep phenotyping of their later disease development, treatment, and prognoses. dbTMM provides a research and development platform that enables cohort stratification, deep phenotyping and prospective analyses of large genomic cohort data.

## Materials and methods

### Cohort studies

As described above, we have conducted two prospective cohort studies: the TMM CommCohort study and the TMM BirThree Cohort study. The TMM CommCohort study targets adult individuals (aged 20 years and older) and recruited a total of 84,073 participants from 2013 to 2016. On the other hand, the TMM BirThree Cohort study targets pregnant women and their families, including the fathers, grandparents and other family members of the fetuses. This study recruited a total of 73,529 participants, including 32,086 children, from 2013 to 2017. In both cohorts, we have collected the following data: (1) demographic data (gender, age, address); (2) laboratory test data (blood and urine test data); (3) questionnaire data on sociodemographic factors, lifestyle habits, and medical history; (4) physiological data, including height, weight, body composition, blood pressure or estimated central aortic blood pressure, heart rate, carotid ultrasound imaging findings, magnetic resonance imaging (MRI) findings, respiratory function parameters, respiratory impedance, calcaneal ultrasound bone mineral density, leg extension strength, grip strength, oral examination results, hearing acuity, eye examination findings, home-measured blood pressure, the number of steps per day, waist circumference, visceral fat measured by bioelectrical impedance analysis, electrocardiography findings, pulse wave velocity, and flow-mediated dilation (see Table 3A/3B^5^ for measurement methods); and (5) clinical data. The exact set of variables depended on where participants underwent their health examinations.

### Biospecimen collection and storage

In the cohort studies, we collected 34 mL of blood into four distinct types of tubes: (1) EDTA-2Na tubes for plasma and buffy coat collection, (2) heparin tubes for peripheral blood mononuclear cell (PBMC) collection, (3) two plain tubes with a separation gel for serum collection and clinical biochemical analysis, and (4) sodium fluoride tubes for blood glucose level analysis. We also collected 3–10 mL of urine from adult participants. We then stored buffy coats, plasma, serum, urine and breast milk in the Brooks BioStore system at −80 °C. In addition, we manually isolated mononuclear cells from peripheral blood or cord blood, and PBMCs and cord blood mast cells (CBMCs) were stored below −180 °C in a liquid nitrogen storage system for long-term storage. We established Epstein-Barr virus (EBV)-transformed B lymphocyte cell lines and activated T lymphocyte cell lines, which were also stored in a liquid nitrogen storage system. Genomic DNA was isolated from frozen buffy coats of peripheral or cord blood or from saliva. Isolated DNA was suspended in 350 µL of TE buffer, and the concentration was adjusted to 50 ng/µL using an automated liquid handling system. DNA quality control (QC) assays were performed using a NanoDrop 2000 (Thermo Fisher Scientific). The adjusted DNA samples were stored in a Brooks SampleStore system at 4 °C. All biospecimen information was managed by using a laboratory information management system (LIMS).

### Genome and multiomics analyses

For genomic analysis, we performed whole-genome sequencing (WGS) with high coverage (32x per sample) and completed deep WGS analysis of 8380 individuals to cover single nucleotide polymorphisms (SNPs). We also performed Japonica array genotyping. We have developed a custom SNP array for the Japanese population, called Japonica arrays^16^, using our whole-genome sequence data. The Japonica array provides better imputation performance for Japanese individuals than the existing commercially available SNP arrays. We completed Japonica array genotyping of 150,000 individuals.

In multiomics analysis, we performed nuclear magnetic resonance (NMR) spectroscopy and nontarget mass spectrometry (MS) to analyze plasma metabolites. For NMR spectroscopy analysis, metabolites were extracted from 200 μl of plasma and suspended in sodium phosphate buffer. All NMR experiments were performed at 298 K (25 °C) on a Bruker 600 MHz spectrometer^17^. For mass spectrometry analysis, metabolites were extracted from a 50-μl plasma sample. MS analysis was performed by liquid chromatography–mass spectrometry (LC–MS) systems^18^.

### Database servers

The database is hosted on 18 servers, each running CentOS Linux release 7.6 with Xeon (12 core) x 2 sockets, with 128 GB of RAM, a 1 TB solid-state drive, a 300 GB hard drive, and 2 TB FC-SAN disk space. Among these servers, 16 are used as MySQL 5.6 database servers, and 2 are used as Apache 2.4 and Tomcat 7.0 servers. We use an InnoDB MySQL storage engine to store all user activity occurring inside a transaction, as we have needed to log all data access to enable us to specify an outflow source in the event of a data leak.

### Database framework

The database system was developed using the Java Development Kit (JDK) and Seasar2. Seasar2 is an open-source framework and enables a system to easily change specifications by using configuration files. The system uses MySQL version 5.6 as a relational database system, and for MySQL database connection, the Java library MySQL Connector/J 5.1 is used. The system uses jQuery for the client side and enables dynamic content of the web interface. It is installed using Apache 2.4 and Tomcat 7.0 as a web and application server on CentOS 7.6 and is open to the users. To submit jobs to the Univa Grid Engine (UGE), the system uses the Java library Distributed Resource Management Application API (DRMAA).

## Results and discussion

### Collection of cohort/clinical and genome/multiomics data

**Fig. 1:**
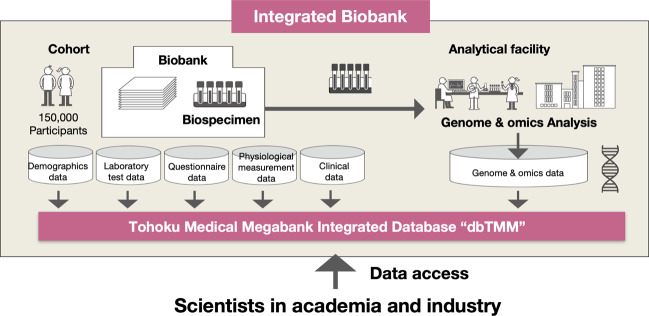
Integrated biobank and integrated database. An integrated biobank is a biobank equipped with analytical facilities and integrating biospecimens and genome/multiomics data derived from them. The integrated biobank conducts genome/multiomics analysis and provides genome/multiomics data in addition to raw specimens. Thus, the TMM biobank retains not only cohort/clinical data but also genome/multiomics data. To incorporate and control all the collected data, we designed and developed an integrated database, which we refer to as “dbTMM”. dbTMM integrates both individual cohort/clinical data and genome/multiomics data from more than 150,000 participants.

Within the TMM project, we developed an “integrated biobank” with both storage and analytical facilities for genome and multiomics analyses under one roof to share not only cohort and clinical data but also genome and multiomics data in addition to biospecimens from participants. Importantly, as shown in Fig. 1, we planned to incorporate all the data into an integrated database, which we refer to as database TMM (dbTMM). We believe that constructing an integrated database is critical for the development and management of a modern integrated biobank.

**Fig. 2:**
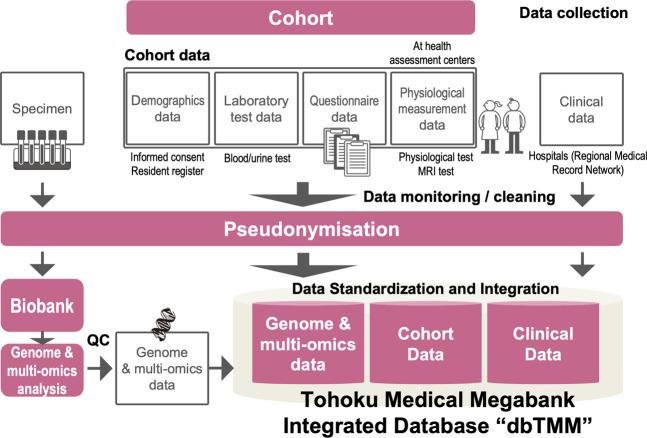
Data collection and the integration of cohort/clinical and genome/multiomics data. We collected biospecimens and their laboratory test data from all participants. We also collected demographic, questionnaire, and physiological data. All the collected cohort data were monitored, cleaned, quality-checked, pseudonymized and stored in the integrated database, dbTMM. Clinical data has been also collected in part from medical records in hospitals. Biospecimens have been obtained from our cohorts and stored in a biobank, and their derived genome and multiomics data, including metabolomic and proteomic data has been stored in dbTMM.

In the TMM CommCohort study and TMM BirThree Cohort study, we first obtained informed consent from all participants and collected both blood and urine specimens along with laboratory test data^4^. We also collected self-reported health status and environmental data through questionnaires (top part of Fig. 2). In addition, we collected physiological data in our health assessment centers (seven community support centers in Miyagi Prefecture and four satellite centers in Iwate Prefecture)^4^. We are also collecting MRI image data from volunteers.

Data monitoring and cleaning are essential for cohort/biobank management. Therefore, we have monitored, cleaned, and quality-checked all collected cohort data (middle part of Fig. 2). Clinical data have also been collected manually from medical records in hospitals by our Genome Medical Research Coordinators (GMRCs) and electronically by means of record linkage. In addition, we are preparing a pipeline to capture clinical and phenotypic data from electronic medical records in hospitals. These data were stored in the integrated database dbTMM after pseudonymization.

In addition to these cohort data, we have collected biospecimens from our cohorts and stored them in our biobank. To construct an integrated biobank, we have been working to transform the biospecimens into analyzed data. To this end, we extracted DNA from blood and rendered the DNA samples for microarray analyses as well as whole- genome sequencing analyses in our analytical facilities. Multiomics analyses, including metabolomics and proteomics analyses, have also been conducted. The analyzed data were quality-checked and stored in dbTMM (lower part of Fig. 2).

### Identifiers and security measures to protect sensitive data

Since the establishment of the TMM project, we have collected various data ranging from cohort and clinical data to genome and multiomics data and efficiently integrated all these data into dbTMM. During these processes, we took special notice of the sensitivity of the data, which met the criteria for strong protection according to our TMM data sharing policy^19^.

To maintain strict security, we decided to manage these data by means of different participant identifiers (IDs). We set up three key IDs: cohort data are under Cohort Data IDs, biospecimen-derived genome/multiomics data are under Biobank IDs, and clinical and phenotypic data are under Clinical Data IDs (lower part of Supplementary Fig.). Access rights to these data are strictly controlled under the terms of service. A user with the privilege of accessing the data is able to look up corresponding tables of IDs. The user can create an integrated view by joining all the tables under the access rights in the database.

It should be noted that the user privilege settings are under strict control. The corresponding tables are kept encrypted and are decrypted only when a privileged user accesses the data. Thus, dbTMM stores various data separately under different IDs, but when it is necessary to integrate genome, cohort and/or clinical data, the user can create an integrated view (Supplementary Fig.).

### Data standardization and quality control

In the construction of dbTMM, we recognized the crucial importance of data preprocessing. To integrate all data in a proper format, we needed to elaborate data standardization and quality control.

For data standardization, it is desirable to describe diagnosed disease names (Dx) by using International Classification of Diseases (ICD)-10 codes^20^, medical prescriptions (Rx) by using ATC (Anatomical Therapeutic Chemical) Classification System codes, and laboratory values by using Japan Laboratory Code version 10 (JLAC10) codes.

In our integrated dbTMM, we have already coded all self-reported disease names into ICD-10 codes. Similarly, we have almost finished the process of coding self-reported medications into ATC codes. However, regarding the laboratory values, we found that the JLAC10 code had a limitation in which the coding results varied from person to person even with comparable laboratory results, making it impossible to ensure that any given value has a unique representation. We are trying to determine how to overcome this limitation (Supplementary Table).

In dbTMM, based on the above codes, we assigned an item code to each item and variable of the cohort and clinical data. There are various types of data in cohort data and clinical data, such as laboratory values and physiological measurements, and as mentioned above, JLAC10 covers the codes for laboratory values, but we prepared an item code to code all the data collected in our project in a common way. For quality control, we appended an error code to each variable, which was detected in the data cleaning and quality check processes.

### Large-scale genome data stored in a distributed database

**Fig. 3:**
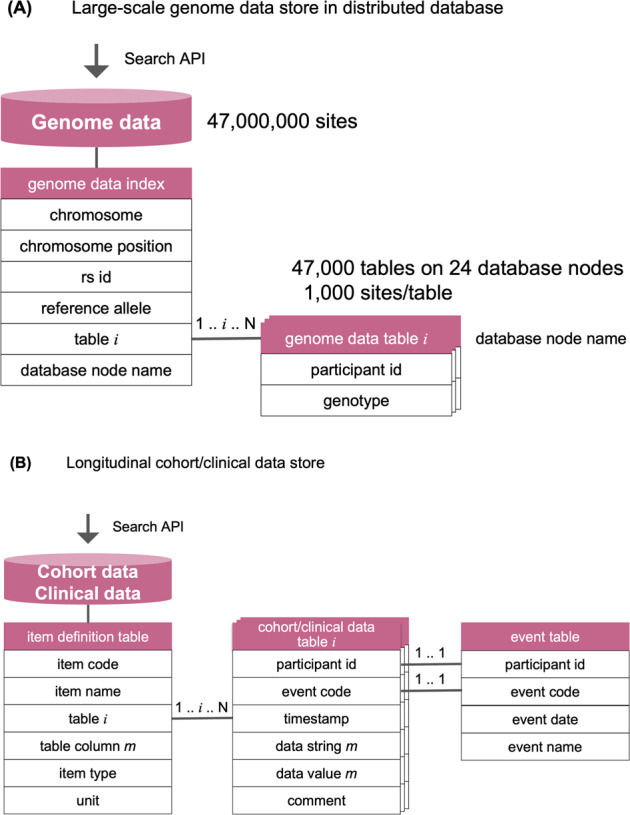
Database design of dbTMM. **A** Large-scale genome data storage in a distributed database. To store large-scale genome data variant by variant over 47 million sites for more than 150,000 participants, dbTMM is designed as a distributed database: two types of MySQL tables were set up, including a genome data index and genome data tables (n tables). The genome data index table represents an index for genotype data in a genome data table. dbTMM accepts search requests by chromosomal position or rs number, and dbTMM looks up the corresponding genotype in a genome data table by using the genome data index table. Each genome data table contains genotype data for each participant for each variant. **B** Longitudinal cohort/clinical data storage. To store longitudinal large-scale cohort and clinical data for more than 150,000 participants, dbTMM is designed as a distributed database: three types of MySQL tables were set up, including an item definition table, data tables (n tables), and an event table. The item definition table represents the definition of each item of cohort data. Each data table contains collected longitudinal data for each participant for each event. The event table contains a field called event code with which it can be referenced by entries in the data tables.

dbTMM is required to store all genome data, variant by variant, across 47 million sites^21^ in 150,000 participants. The data are too large to be stored in one database. To enable users to search large-scale genome data on a variant-by-variant basis, we designed dbTMM as a distributed database (Fig. 3(A)). We stored genome data from 47 million sites in 47,000 tables, each of which covers 1000 sites. We are running the ToMMo supercomputer, consisting of many nodes, and the 47,000 tables are stored in 24 database nodes in a distributed manner.

The dbTMM Search API accepts requests from users to search for variants, which can be specified by chromosomal position or rs number. Upon receiving the search request, the Search API will look up the genome data index table to find the table number where the genotype data of the variant are stored and the database node name where the table is located. The Search API refers the genotype of the variant to the corresponding table and returns the search results very fast.

### Storing longitudinal cohort/clinical data along with cohort study

dbTMM is required to store longitudinal large-scale cohort and clinic/hospital data. We have collected blood/urine specimens and their laboratory test values, along with health status and environmental data self-reported on questionnaires. Approximately 18,000 participants had physiological data, which were taken at baseline and at follow-up examinations every 5 years for the participants who agreed to undergo follow-up. We also collected clinical data from hospitals. Therefore, we need to distinguish data from each visit or event, e.g., a baseline assessment and a 5-year follow-up assessment. To enable users to search longitudinal cohorts and clinical data event by event, we designed dbTMM as a time-course database, providing timestamps and various events (Fig. 3(B)).

A timestamp was used to distinguish the day on which an event (including a visit to the assessment center) took place, and an event code was used to distinguish an event in the life course (including an assessment in the cohort study). Usually, a participant visits the health assessment center once for a single assessment (event); in this case, the participant has cohort data from a single visit as an assessment event. Occasionally, participants interrupted their health assessments due to certain time constraints or bad health conditions, but they usually visited us again to complete their assessment; in this case, the participant had cohort data from two visits as an assessment event. We use a composite primary key consisting of a timestamp and an event code to distinguish the day on which an event occurred. For example, if a subject visits the health assessment center once as a baseline survey, the data with a single timestamp are stored as a single baseline survey event. On the other hand, if a subject visits the health assessment center once as a baseline survey but stops the survey due to illness and comes back again, then the subject is regarded to visit the center twice. Therefore, the data with two timestamps are stored as one baseline survey event.

The dbTMM Search API accepts a request from a user to search for cohort/clinical data, which can be specified by item code. Upon receiving a search request, the Search API will look up the item definition table to find the table number where the cohort/clinical data item is stored. The item definition table represents a definition of an item for cohort data. The Search API refers the cohort/clinical data item to the corresponding table and retrieves its value and event code. Each cohort/clinical data table contains collected longitudinal data for each participant for each event. All events are managed in the event table with the code, date and name of the event for each subject. Finally, the Search API presents users with the results of the search for cohort/clinical data values and events.

### Feature extraction for narrowed populations

By using dbTMM, users can stratify cohorts and then browse both individual cohort/clinical data and genome/multiomics data from the stratified population. dbTMM further allows users to browse features of the stratified population. dbTMM conducts statistical analysis on narrowed-down populations to detect variables that exhibit statistically significant differences between the stratified population and the parent population.

Users are able to identify the characteristic variables, that is, features, in which the stratified population shows a statistically significant difference compared to the population. Identified feature variables can help users find ideas for research hypotheses or, in some cases, suggest a hidden bias in the stratified population. However, dbTMM stores so many variables that researchers may not be able to browse them all. Thus, the large-scale database must enable users to extract and present the features of the narrowed population while narrowing down the search.

### Database release

We built a database for each cohort and for each type of recruitment. As described above, we have conducted the CommCohort study and BirThree Cohort study. The CommCohort study has two major types of recruitment: one is a survey based on municipal health checkups, while the other is a survey based on an assessment center^5^. Regarding the assessment center, there are seven community support centers and four satellite centers.

In each cohort and each recruitment type, basic genome, multiomics, and cohort data were collected from participants. The categories, subcategories, current number of data entries, number of variables, number of participants, and number of releases are shown in Table 1. The data in this table represent a snapshot of the database as of Oct 20, 2019. dbTMM stores hundreds of millions of genome, multiomics, and cohort data; the total number of data entries is over 1.3 trillion.

**Table 1:** Database statistics: data category, number of data entries, variables, and participants.

Data category	Data subcategory	Number of data entries	Number of variables	Number of participants
Demographics data		403,073	9	165,511
Health data	Laboratory test	7,281,074	655	132,913
	Questionnaire	55,201,730	2931	182,903
	Health-checkup	1,339,470	39	70,888
	Physiological test	5,577,101	505	26,003
	Magnetic resonance imaging	6,897,799	1614	4279
	Cognitive/psychological test	1,473,234	356	4279
Clinical data	Medical record data	5,138,301	1161	22,797
Genome data	Whole genome data	183,117,102,675	55,238,945	3315
	SNP array genotype data	2,854,495,579,124	86,836,730	65,744
Omics data	NMR metabolome data	138,564	37	3761
	MS metabolome data	382,113	415	2900
	Proteome data	88,685	577	497

The collected data comprise 12 subcategories, i.e., demographics, laboratory tests, questionnaires, health checkups, physiological tests, MRI, cognitive/psychological tests, whole-genome sequences, SNP array genotypes, NMR metabolome analysis, MS metabolome analysis, and proteome analysis. All the collected data are released for each cohort and each recruitment type.

### Database performance and scalability

TMM cohort studies started in 2013, and dbTMM started in 2016. We first released the whole-genome sequence data of 1000 participants in the CommCohort study along with their basic cohort data. The coverage of dbTMM has been growing, and data in a number of subcategories have been integrated on all 150,000 participants.

**Fig. 4:**
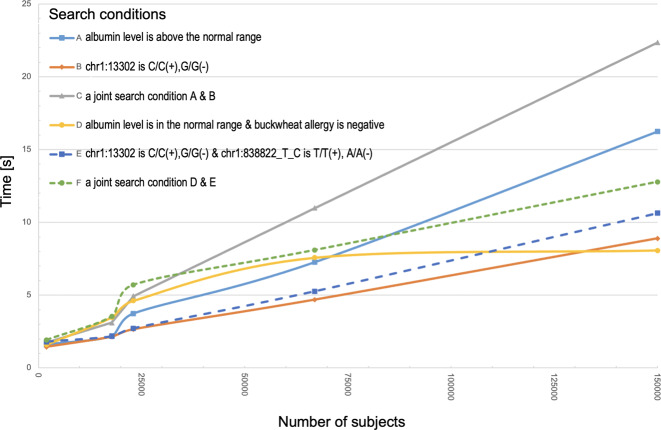
Database performance and scalability of dbTMM. Database performance for five search conditions and database scalability for up to 150,000 participants. Search condition A is for cohort data in which the albumin level is above the normal range. Search condition B is for genome data in which the genotype for chr1:13302 is C/C(+),G/G(–). Search condition C is a combination of search conditions A and B. Search condition D is for cohort data in which the albumin level is in the normal range and buckwheat allergy is negative. Search condition E is for genome data in which the genotype for chr1:13302 is C/C(+),G/G(–) and the genotype for chr1:838822_T_C is T/T(+), A/A(–). Search condition 6 is a combination of search conditions D and E. Note that the performance of dbTMM scales linearly in all search conditions.

In Fig. 4, we show the performance and scalability of the database for the 150,000 participants. It should be noted that the performance of dbTMM scales linearly in all search conditions examined, including the following: the albumin level is above the normal range (A), chr1:13302 is C/C( + ),G/G(–) (B), a combination of search conditions A & B (C), albumin level is in the normal range & buckwheat allergy is negative (D), chr1:13302 is C/C( + ),G/G(–) & chr1:838822_T_C is T/T( + ), A/A(–) (E), and a combination of search conditions D & E (F). These results provide assurance that dbTMM can be searched efficiently and swiftly even with a large number of participants, demonstrating that dbTMM retains sufficient performance for the searching of 150,000 participants’ data. However, the reason for the presence of a small deviation at ~25,000 remains to be clarified.

### Database access and search

To access dbTMM, users need to register for a user account (for information on registration for a user account, see https://www.megabank.tohoku.ac.jp/researchers/ dist#dbtmm). After the registered user account is authenticated and the user agrees to the terms of use, a user can start accessing dbTMM. Of the many important conditions with which the users need to agree or comply, we emphasize that the users should not attempt to reidentify our participants. Once registered and authenticated, users can access dbTMM to seek data for research and development.

**Fig. 5:**
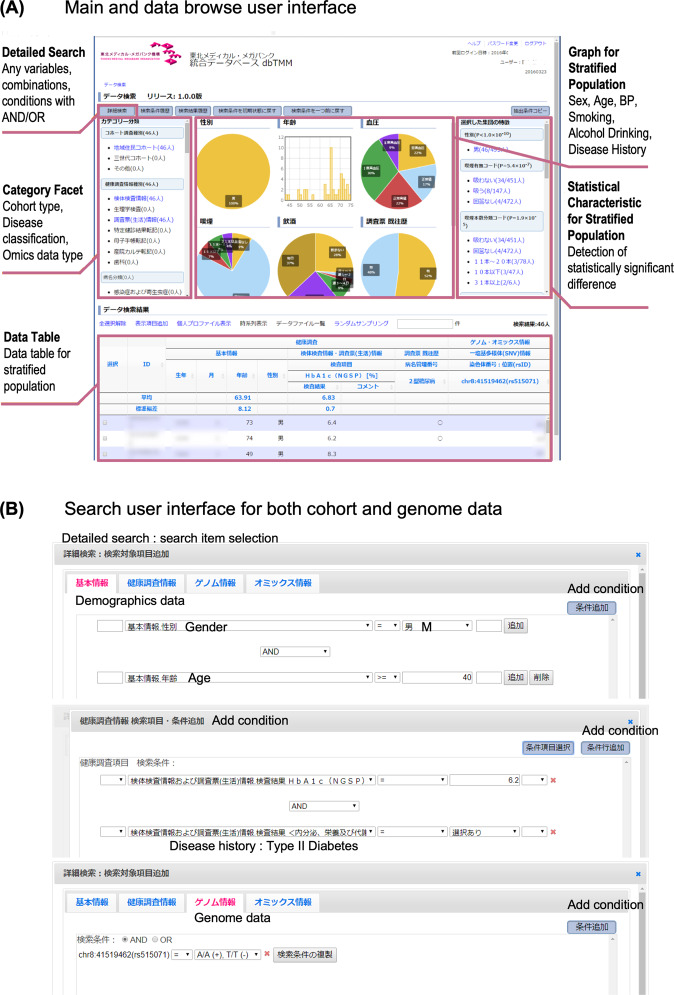
User interface of dbTMM. **A** Main and data browsing user interface. The top center panel is a panel of graphs of epidemiological data. The upper left panel is a search panel for categorical facets. The lower panel is a data table panel with search results. The upper right panel is a panel displaying the detected features in the narrowed-down population. **B** The search user interface for both cohort and genome data. The typical search interface is shown. Users can set search conditions for cohort data and genome data.

Users can access dbTMM with the user interface, as shown in Fig. 5(A). The top center is a panel that shows graphs of epidemiological data for gender, age, blood pressure, smoking, alcohol consumption, and medical history. The upper left is a search panel for categorical facets, allowing the user to search by cohort type, the type of cohort data or genome/multiomics data, disease name, and history. The lower panel is a data table panel with search results, showing the data of subjects in the cohort that match the search criteria. The upper right panel displays the detected features that are significantly different in the narrowed-down population matching the search criteria compared with the entire population.

Users can search all the genomic variant, multiomics, cohort and clinical data in dbTMM, which enables users to precisely stratify our cohort population by a combination of these data. Users can set a search condition for cohort data, for example, gender is male, age is over 40 years, HbA1c is over 6.2, alcohol drinking is present, and a history of type II diabetes history is present. Similarly, as shown in Fig. 5(B), users can also set search conditions for genome data, specifying, for example, that the genotype for rs515071 should be A/A( + ),T/T(-).

As a result, users can, for example, precisely narrow the subjects down to 46 participants for a diabetes study considering genetic background (Fig. 5(A); Data Table). Thus, users are able to access dbTMM and search for the data that are needed for research and development. The next step is that the user applies for data use. If the application is approved, the user can access the data extracted from dbTMM, and the user can then carry out the approved research.

## Conclusion

We developed dbTMM, a database that integrates both individual cohort/clinical data and genome/multiomics data from 157,191 participants in the Tohoku Medical Megabank project. To our knowledge, dbTMM is the first database to store individual whole-genome data on a variant-by-variant basis and cohort/clinical data for more than one hundred thousand participants in a prospective cohort study. dbTMM enables users to stratify the cohort by both genetic and environmental factors and provides a research and development platform. The platform enables prospective analysis of large-scale data from genome cohorts, which leads to the establishment of a new disease concept in genomic medicine for multifactorial diseases caused by the complex interaction of genetic and environmental factors.

## Supplementary information

Supplementary Figure

Supplementary Table

## Appendix

Including the initially mentioned investigators, the Tohoku Medical Megabank Project Study Group members as of 1 September 2018 are as follows: ***Tohoku University Tohoku Medical Megabank Organization (ToMMo)***, ***Principal Investigator:*** Masayuki Yamamoto. ***Study Group Members:*** Michiaki Abe, Yayoi Aizawa, Yuichi Aoki, Koichi Chida, Inaho Danjoh, Shinichi Egawa, Ai Eto, Takamitsu Funayama, Nobuo Fuse, Yohei Hamanaka, Yuki Harada, Hiroaki Hashizume, Shinichi Higuchi, Sachiko Hirano, Takumi Hirata, Masahiro Hiratsuka, Atsushi Hozawa, Kazuhiko Igarashi, Jin Inoue, Noriko Ishida, Naoto Ishii, Tadashi Ishii, Mami Ishikuro, Kiyoshi Ito, Sadayoshi Ito, Maiko Kageyama, Fumiki Katsuoka, Hiroshi Kawame, Junko Kawashima, Masahiro Kikuya, Kengo Kinoshita, Kazuyuki Kitatani, Tomomi Kiyama, Hideyasu Kiyomoto, Tomoko Kobayashi, Eiichi Kodama, Mana Kogure, Kaname Kojima, Sachie Koreeda, Seizo Koshiba, Shihoko Koyama, Hisaaki Kudo, Kazuki Kumada, Shigeo Kure, Miho Kuriki, Shinichi Kuriyama, Yoko Kuroki, Norihide Maikusa, Satoshi Makino, Hiroko Matsubara, Hiroyuki Matsui, Hirohito Metoki, Takahiro Mimori, Naoko Minegishi, Kazuharu Misawa, Masako Miyashita, Satoshi Mizuno, Hozumi Motohashi, Ikuko N. Motoike, Satoshi Nagaie, Masato Nagai, Fuji Nagami, Masao Nagasaki, Sachiko Nagase, Naoki Nakamura, Tomohiro Nakamura, Naoki Nakaya, Keiko Nakayama, Akira Narita, Ichiko Nishijima, Takahiro Nobukuni, Kotaro Nochioka, Taku Obara, Soichi Ogishima, Noriaki Ohuchi, Gervais Olivier, Noriko Osumi, Hiroshi Otsu, Akihito Otsuki, Daisuke Saigusa, Sakae Saito, Tomo Saito, Masaki Sakaida, Mika Sakurai-Yageta, Yuki Sato, Yukuto Sato, Atsushi Sekiguchi, Chen-Yang Shen, Tomoko F. Shibata, Ritsuko Shimizu, Kazuro Shimokawa, Matsuyuki Shirota, Junichi Sugawara, Kichiya Suzuki, Yoichi Suzuki, Shu Tadaka, Makiko Taira, Takako Takai-Igarashi, Yuji Takano, Yasuyuki Taki, Gen Tamiya, Osamu Tanabe, Hiroshi Tanaka, Yukari Tanaka, Shunsuke Teraguchi, Takahiro Terakawa, Teiji Tominaga, Hiroaki Tomita, Akito Tsuboi, Naho Tsuchiya, Ichiro Tsuji, Masao Ueki, Akira Uruno, Nobuo Yaegashi, Junya Yamagishi, Yumi Yamaguchi-Kabata, Chizuru Yamanaka, Riu Yamashita, Jun Yasuda, Junji Yokozawa, and Kazunori Waki. ***Iwate Medical University Iwate Tohoku Medical Megabank Organization (IMM)***, ***Principal Investigator:*** Makoto Sasaki. ***Study Group Members:*** Junko Akai, Ryujin Endo, Akimune Fukushima, Ryohei Furukawa, Tsuyoshi Hachiya, Kouhei Hashizume, Jiro Hitomi, Yasushi Ishigaki, Shohei Komaki, Yuka Kotozaki, Takahiro Mikami, Motoyuki Nakamura, Naoyuki Nishiya, Satoshi Nishizuka, Yoko Nomura, Kuniaki Ogasawara, Hideki Ohmomo, Shinichi Omama, Ryo Otomo, Kotaro Otsuka, Kotaro Oyama, Kiyomi Sakata, Ryohei Sasaki, Mamoru Satoh, Namie Sato, Atsushi Shimizu, Yu Shiwa, Yoichi Sutoh, Nobuyuki Takanashi, Noriko Takebe, Fumitaka Tanaka, Ryoichi Tanaka, Kozo Tanno, Tomoharu Tokutomi, Kayono Yamamoto, and Fumio Yamashita.
