# Supplementary figures and images for: dbTMM: an integrated database of large-scale cohort, genome and clinical data for the Tohoku Medical Megabank Project
Source: Hum Genome Var. 2021 Dec 10;8:44. doi: 10.1038/s41439-021-00175-5 (PMC8660797; doi:10.1038/s41439-021-00175-5)

Supplementary Fig. **IDs and security measures to protect sensitive data**

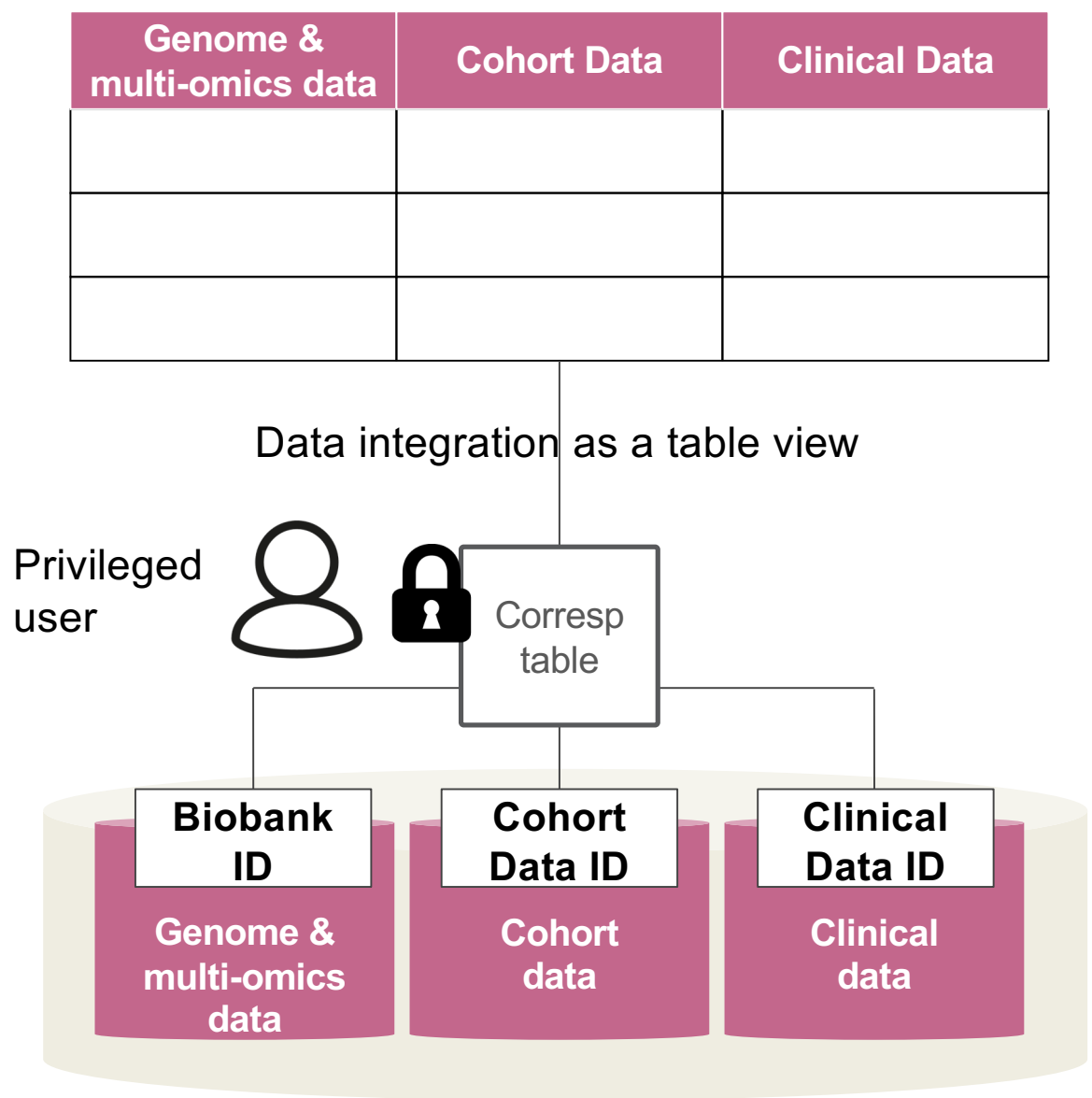

Supplement: Supplementary file 1 — Supplementary Figure [file 41439_2021_175_MOESM1_ESM.pdf]
