# Supplementary material for: dbTMM: an integrated database of large-scale cohort, genome and clinical data for the Tohoku Medical Megabank Project
Source: Hum Genome Var. 2021 Dec 10;8:44. doi: 10.1038/s41439-021-00175-5 (PMC8660797; doi:10.1038/s41439-021-00175-5)
Supplement: Supplementary file 2 — Supplementary Table [file 41439_2021_175_MOESM2_ESM.pdf]

Supplementary Table Data type and standard

Data type	Standard	Status
Disease name	ICD (International Classification of Diseases) 10	Applied
Medical prescription	ATC (Anatomical Therapeutic Chemical) Classification System	Applicable soon
Laboratory values	JLAC (Japan Laboratory Code Version) 10	Not yet applied
